# Microaneurysm segmentation under out-of-domain generalization: from diabetic retinopathy to leukemic retinopathy

**DOI:** 10.1186/s40942-026-00844-z

**Published:** 2026-05-16

**Authors:** Edgar Platas-Campero, Raquel Díaz-Hernández, Anabel Sánchez-Sánchez, Saúl Zapotecas-Martínez, Nohemi Sánchez-Medel, Sashwanthi Mohan, Jamyl Habib-Castillo, Leopoldo Altamirano-Robles

**Affiliations:** 1 National Institute of Astrophysics, Optics and Electronics (INAOE), Puebla, México; 2 Specialist Ophthalmologist-Cataract and Vitreoretina, Medcare Eye Centre, Al Safa, Dubai, United Arab Emirates; 3 Specialist Ophthalmologist, HABIB Ophthalmology Center, Monterrey, México

**Keywords:** Semantic segmentation, Out of domain generalization, Microaneurysm, Diabetic retinopathy, Leukemic retinopathy.

## Abstract

**Purpose:**

To propose inter-disease out-of-domain generalization (OODG) across retinal diseases for microaneurysm (MA) segmentation using a deep-learning model trained and validated on diabetic retinopathy (DR) and qualitatively evaluated on leukemic retinopathy (LR).

**Methods:**

A U-Net based segmentation model was trained using the IDRiD dataset, which comprises 81 DR images, using only MA annotations. The images were split into patches, and a statistical filtering step was applied to retain only structurally homogeneous patches. The study was organized in two phases: in Phase I, the U-Net was trained and evaluated using DR patches; in Phase II, the model was tested directly, without retraining, on LR images. Finally, MA segmentations were subjected to qualitative assessment by clinical specialists.

**Results:**

The proposed U-Net achieved an IoU of 0.842, a Dice score of 0.914, an accuracy of 0.998, and a validation loss of 0.120.

**Conclusion:**

The results suggest that knowledge learned from DR can generalize effectively to a related clinical context such as LR, opening the possibility of reusing models in diseases with similar structures and lesion patterns.

## Introduction

Microaneurysms (MAs) are focal saccular dilations of retinal capillaries, caused by weakening of the vascular wall  [1]. In color fundus photographs, they usually appear as very small dark-red dots; reported diameters range from approximately 10 to $100\,\mu\,\textrm{m}$. Their detection is challenging due to their small size and low contrast against the background, and they have been reported to account for ~1% of the visible content in an image  [2–4].

These retinal lesions are highly relevant because they are considered early indicators of several disorders, particularly diabetic retinopathy (DR), as they may appear before the onset of visual symptoms  [5]. In addition, they serve as activity and progression markers, since their number, distribution, and dynamics of appearance or disappearance are closely correlated with the evolution of microvascular damage  [6]. In this sense, DR is useful for studying microvascular lesions such as MAs due to their high frequency and the availability of widely standardized clinical criteria. In the non-proliferative stage, MAs are the hallmark finding and an early indicator of microvascular damage; therefore, their timely detection and the analysis of their turnover rate are key to anticipating progression and reducing the risk of vision loss, as they act as biomarkers of vascular stability and therapeutic response  [7–9].

MAs can be observed in different conditions such as hypertensive retinopathy (HR)  [10], leukemic retinopathy (LR)  [11], Kawasaki disease (KD)  [12], and ocular ischemic syndrome (OIS)  [13]. This highlights the importance of identifying these retinal lesions and creates an opportunity to develop a model that can be trained on one disease and evaluated on another. In the case proposed here, the model is trained on DR and also evaluated on LR, which was selected particularly due to limited data availability. Consequently, the problem is formulated as inter-disease generalization and is framed within out-of-domain generalization (OODG).

OODG refers to a model’s ability to maintain performance when evaluated on unseen data despite distribution shifts  [14], for example, differences in acquisition conditions, variations in lesion patterns and distributions, or increased retinal heterogeneity. OODG in medical imaging has been addressed through multiple methodological variants. In the field of data augmentation, BigAug demonstrated that massive stacked augmentation can reduce degradation in organ segmentation for MRI and ultrasound from 39.3% to 11.6% without accessing the target domain  [15]. From an adversarial domain adaptation perspective, methods such as StarGAN have managed to recover up to 25% of the performance lost due to institutional shifts in chest X-rays, introducing the concept of domain spread as an a priori measure of the expected performance of a model when deployed in external domains  [16]. The integration of clinical ontologies into neuro-symbolic frameworks with ViT models has shown improvements of up to 4.2% in cross-domain generalization scenarios for DR classification, arguing that retinal lesions act as domain-invariant features due to the stability of their clinical definitions  [17]. Regarding unsupervised domain adaptation, frameworks such as DDS-UDA have reduced degradation in cross-dataset segmentation of the optic disc and cup in fundus images by integrating bidirectional cross-domain consistency and intra-domain pseudo-label learning  [18]. Within the retinal domain specifically  [19] demonstrated that multi-source adaptation methods achieve competitive performance in cross-dataset DR classification (DDR, IDRiD, Messidor to APTOS 2019) without access to the original source data, using only pre-trained models and unlabeled target images.

Assessing OODG is clinically relevant because it helps estimate model robustness while simultaneously addressing two practical limitations: the scarcity of annotations for low-prevalence pathologies such as LR and the limited feasibility of deploying and maintaining multiple specialized models when associated disorders coexist.

In line with this need to evaluate out-of-domain robustness, several studies have addressed MA segmentation associated with DR using U-Net–type architectures trained on the IDRiD dataset  [20–27]. However, evidence of their performance when applied outside the DR domain remains limited. In this context, this work proposes the direct application, without retraining or domain adaptation, of an MA segmenter trained on DR and evaluated on LR as a case of inter-disease generalization, complemented with qualitative validation by clinical specialists.

## Methodology

**Fig. 1:**
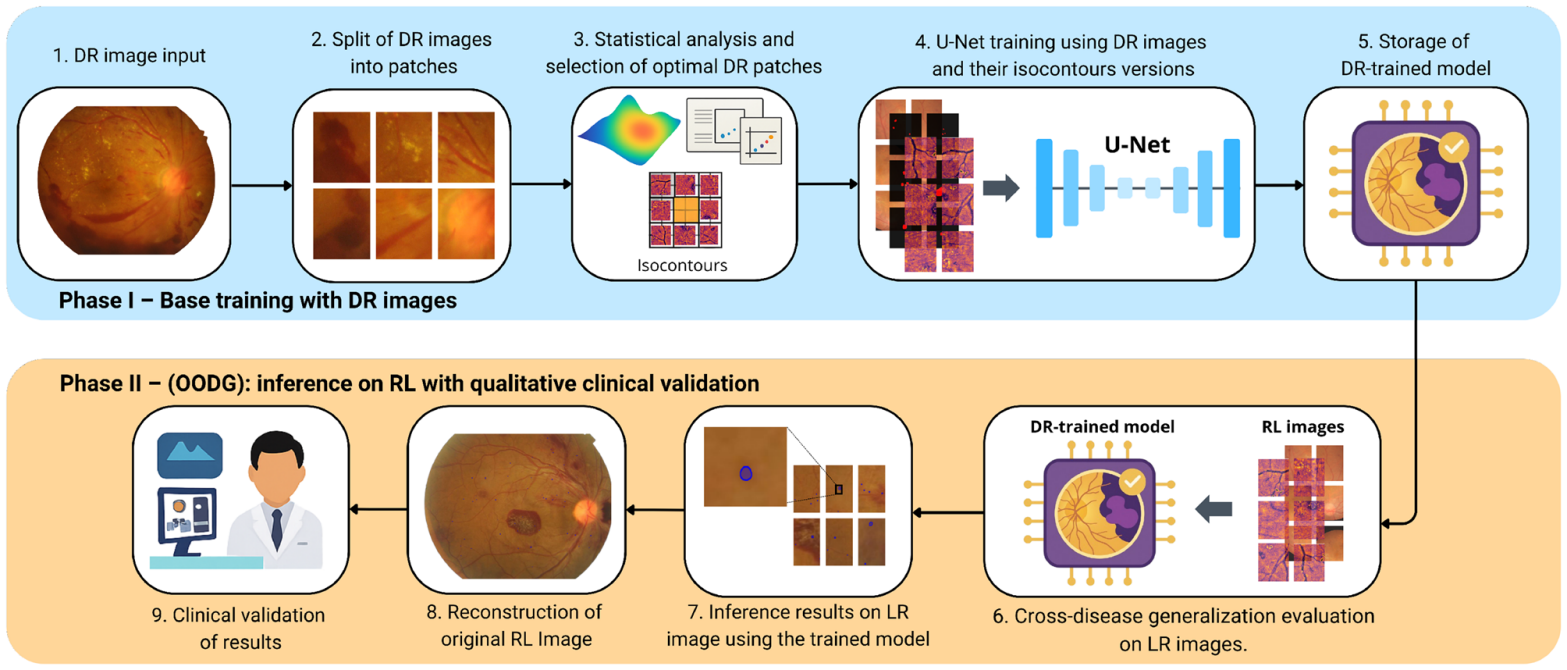
Proposed methodological scheme for segmentation of MA

The proposed methodology starts with images corresponding to DR cases, as illustrated in Fig. 1. To promote local analysis of regions of interest and reduce computational cost, each image is divided into fragments (patches). In addition, a complementary isocontour channel is generated from each patch, designed to enhance subtle intensity variations associated with microvascular structures. Subsequently, a statistical analysis is performed to identify homogeneous patches and retain only those that provide more stable training conditions, reducing strong variations associated with background, illumination, or non-informative content.

In Phase I, a U-Net model was trained and evaluated using DR patches, leveraging the MA reference masks from the IDRiD dataset. In addition, an isocontour channel was generated and incorporated as complementary information to highlight subtle intensity variations and edges associated with these lesions. This stage enabled hyperparameter tuning, overfitting control, and the establishment of a robust baseline model for MA segmentation.

In Phase II, the previously trained model was validated directly, without retraining, on LR images to automatically segment MAs under an inter-disease generalization setting. To this end, LR images were split into patches and, for each patch, the isocontour channel was generated and incorporated as an additional input, consistent with the Phase I configuration. Patch-level predictions were then reassembled to reconstruct the full segmentation mask at the original image scale.

Subsequently, the results were subjected to qualitative assessment by clinical specialists, who reviewed the spatial coherence of the segmentations, their anatomical consistency, and their potential usefulness as diagnostic support.

To contextualize the rigor of this evaluation, the clinical judgment independently performed by the experts is based on four morphological characteristics described in the literature: 1) a round-to-oval, symmetric lesion without irregular projections  [28]; 2) a uniform dark red color, visible as a discrete dot  [29]; 3) sharp margins without peripheral blurring  [30]; and 4) localization as protrusions connected to the capillary wall, predominantly in the deep capillary plexus  [31].

Based on this clinical framework, the instrument evaluates these morphological characteristics using a Likert scale, grounded in the clinical qualitative evaluation protocol validated by  [32]. For instance, the Clinical Precision scale defines its maximum level when segmentations clearly align with the clinical appearance of a microaneurysm. Likewise, the dimension assessing the Severity of Confusion directly measures whether the algorithm failed regarding morphological boundaries. Finally, the Confidence Level item reflects the specialist’s certainty regarding the exact anatomical correspondence of the segmentation.

Therefore, by structuring the evaluation into ordinal scales (Likert) based on these implicit clinical criteria, the protocol provides a statistically valid foundation to determine whether the segmentation model possesses diagnostic utility in the identification of these lesions.

The evaluation panel consisted of four ophthalmology specialists from different countries and institutions, with no professional relationship between them. All evaluations were conducted completely independently, without prior calibration sessions, without post-evaluation consensus sessions, and without communication between evaluators during the process. This geographical and methodological independence prevents anchoring bias (groupthink) and allows us to state that the algorithm is applicable.

Regarding the possibility of expanding the design, a manual segmentation stage by the specialists was added. This was done with the purpose of having a quantitative evaluation that allows for a comparison between the annotations performed by the experts and the identification carried out by the model.

### Dataset: IDRiD and patch image splitting

The Indian Diabetic Retinopathy Image Dataset (IDRiD)  [33] was created to develop and evaluate fundus image analysis algorithms, with an emphasis on early DR and macular edema detection.

This dataset consists of color images (jpg) and pixel-level annotations provided as binary masks (tif) for typical lesions, such as MAs, hemorrhages (HE), hard exudates (EX), soft exudates (SE), and the optic disc (OD). The images were acquired with a Kowa VX-10 camera, with a 50^∘^ field of view, centered near the macula, at a resolution of 4288 × 2848 pixels; each .jpg file is approximately 800 KB.

The dataset provides 81 masks for MA, 81 for EX, 80 for HE, and 40 for SE; these values correspond to the number of binary masks per lesion type. In this study, only the MA class was used for the segmentation task.

### U-Net network architecture and configuration

We implemented an architecture based on the U-Net convolutional neural network, originally proposed by Ronneberger et al.  [34], specifically modified to process images with multiple input channels.

The overall structure of this network consists of an encoder with five successive double-convolution stages, an intermediate representation stage called the bottleneck, and a symmetric decoder with five stages that progressively recover the original spatial resolution.

Each encoder block is composed of a DoubleConv operation, which includes two consecutive 2D convolutions with 3 × 3 kernels, each followed by batch normalization (BatchNorm2d) and a ReLU activation. As encoding progresses, spatial resolution is reduced via 2 × 2 MaxPooling layers, while the number of feature-map channels increases following the sequence: 64 $\rightarrow$ 128 $\rightarrow$ 256 $\rightarrow$ 512 $\rightarrow$ 1024.

In the bottleneck stage, a DoubleConv block is applied to further process the most compressed representation, increasing the depth from 1024 to 2048 feature channels and enabling the extraction of highly abstract features.

During decoding, a symmetric procedure is used to restore the original spatial resolution via bilinear interpolation (upsampling), followed by 1 × 1 convolutions to adjust the number of channels. At each decoder level, the upsampled feature maps are concatenated with the corresponding encoder features through skip connections and processed with a DoubleConv block, facilitating the recovery of fine spatial details for pixel-level segmentation.

## Experiments and results

### Quantitative analysis of domain divergence between DR and LR datasets for OOD designation

To characterize the domain shift, per-channel intensity statistics (mean, standard deviation, and contrast ($\sigma/\mu$) were calculated, along with Jensen-Shannon (JS) and Kullback-Leibler (KL) divergences. These were computed over normalized 256-bin histograms constructed from the pooling of all pixels in each domain. Intensity statistics and JS divergence in image space followed established practices for quantifying domain shift in medical imaging  [35]; KL divergence was reported as a complementary measure  [36]. JS was selected as the primary metric due to its symmetry and bounded range [0, 1], making it more robust than KL for comparisons between heterogeneous distributions. Given the small number of available LR images (*n* = 2), a pooled population distribution approach was adopted instead of the pair-wise calculation proposed in previous works  [35].

Statistical Analysis of Intensity and Contrast: The results reflect substantial differences in the visual composition of both domains Table 1. In grayscale, the DR domain (*n* = 81) presented a mean intensity of 64.5, compared to 84.3 in LR (*n* = 2), indicating that leukemic images are on average brighter and more homogeneous. Regarding color balance, mean values in DR were R:118.3, G:58.0, and B:17.1, while in LR they were R:144.7, G:79.8, and B:28.5. These differences correspond to the distinct vascular and fundus manifestations of each pathology. Contrast ($\sigma/\mu$) was 0.6908 in DR versus 0.3774 in LR, reflecting the more uniform nature of the leukemic images.

**Table 1:** Per-image intensity statistics for DR and LR datasets

Image	µ_R_	µ_G_	µ_B_	µ_gray_	Contrast ($\sigma/\mu$)
*Global averages*					
DR	118.3	58.0	17.1	64.5 ± 10.6	0.6908
LR	144.7	79.8	28.5	84.3 ± 4.4	0.3774

μR, μG, μB: mean pixel intensity per RGB channel (0–255). μgray: mean grayscale intensity ± standard deviation across images. Contrast: internal contrast ratio computed as σ/μ of the grayscale channel

Domain Divergence Metrics: JS divergence yielded values exceeding 0.32 across all channels: JS = 0.3235 (R), JS = 0.3584 (G), JS = 0.3972 (B), and JS = 0.3891 (grayscale) Table 2. These are classified as HIGH domain shift according to the JS > 0.30 criterion. Complementary KL divergence (DR to LR) confirmed this separation with values of 0.8641 (R), 0.9474 (G), 0.9606 (B), and 1.3130 (grayscale)  [36]. Together, these values quantify a domain shift between both populations  [35].

**Table 2:** Jensen-Shannon and Kullback-Leibler divergence per channel between DR and LR domains

Channel	JS divergence	KL divergence (DR→LR)
R	0.3235	0.8641
G	0.3584	0.9474
B	0.3972	0.9606
Gray	0.3891	1.3130

JS divergence ranges from 0 (identical distributions) to 1 (completely different distributions). KL divergence is computed asymmetrically from DR to LR

**Fig. 2:**
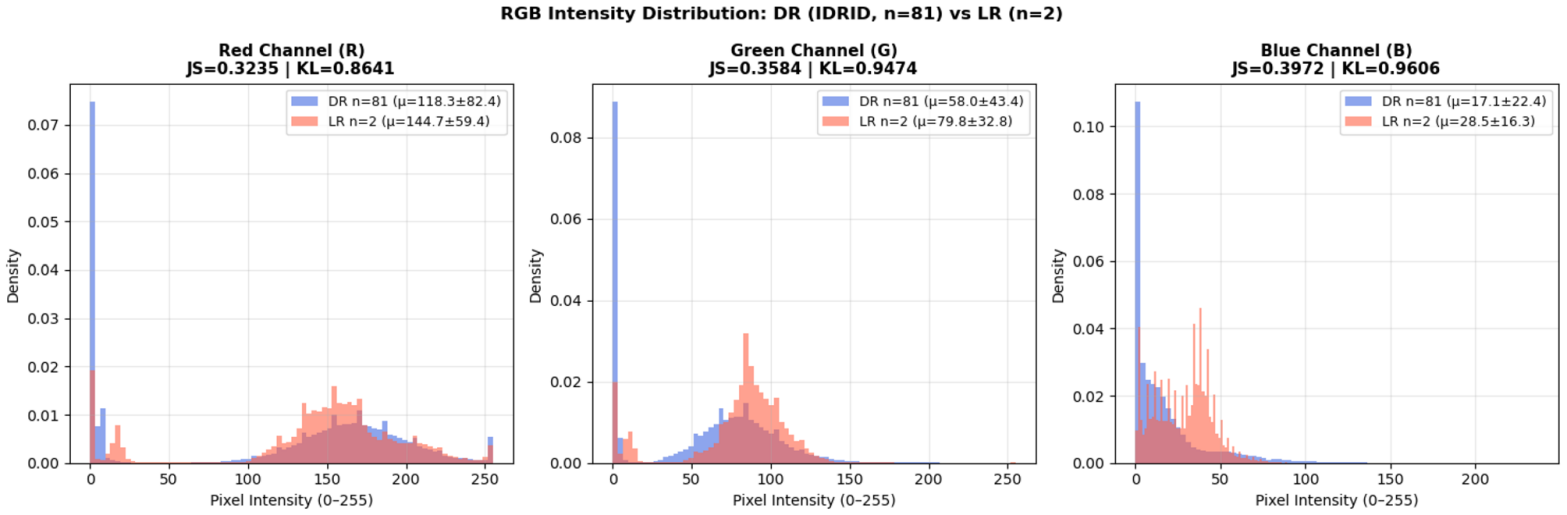
RGB intensity distributions across DR and LR domains

Clinical Interpretation of OOD: These data confirm that the distribution shift operates at the level of the global image context: ocular fundus, color balance, and overall contrast, as shown in Fig. 2. Nevertheless, the robustness of the model lies in the fact that the target lesion, the microaneurysm, possesses a morphological identity independent of the condition. Having internalized the geometric features of MAs in an extensive domain (DR, *n* = 81), the model generalizes to the LR domain by localizing a known anatomical pattern within a different clinical context.

### Distribution of generated patches

**Fig. 3:**
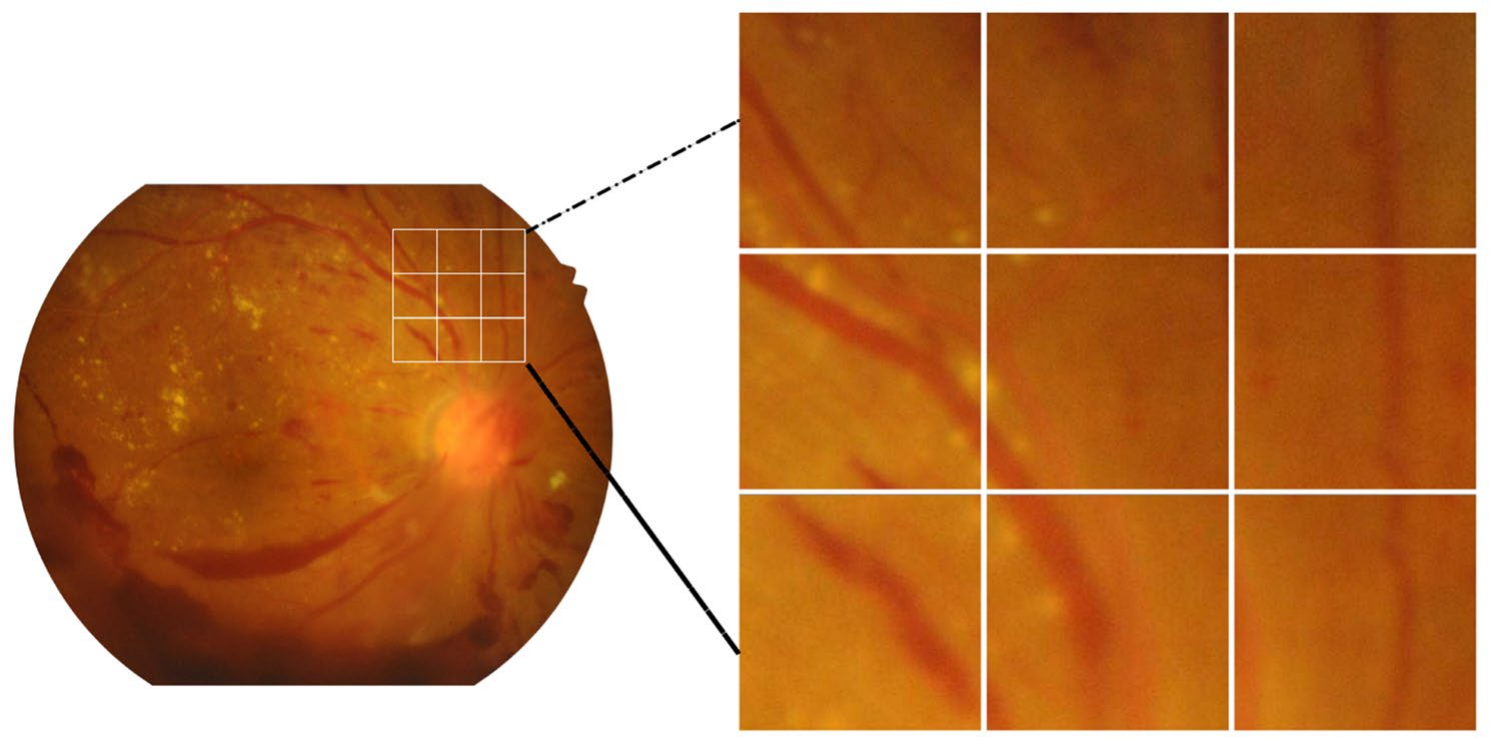
Example of patches sliced from DR images  [33]

Considering the 81 resized original images and their masks, 10,213 patches of 384 × 384 pixels were generated through annotation-guided sampling, as shown in Fig. 3. Instead of using a uniform sliding window, up to 12 patches were extracted for each annotated region by introducing controlled random shifts (jitter of 24 and 48 pixels), while enforcing a minimum distance of 32 pixels between patch centers to avoid duplicates.

In addition, patches without lesion signal were discarded by requiring at least one positive pixel (implemented via a criterion based on the red channel).

With the generated patches, the next step was to perform statistical analyses to evaluate similarity.

### Statistical results for similarity calculation in the dataset

#### 2.5D visualization with Isocontours

**Fig. 4:**
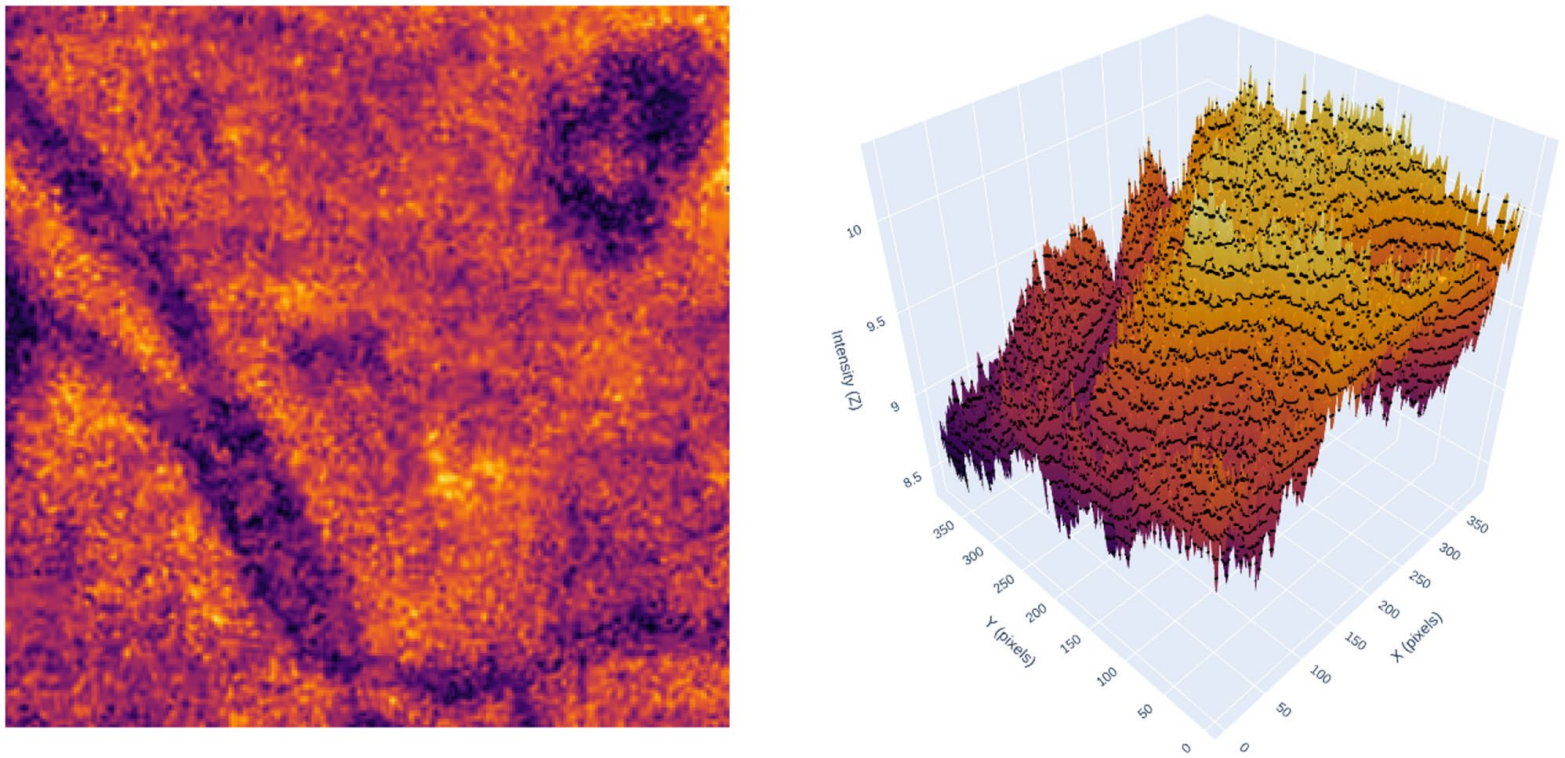
Comparison of patch representations: (left) 2D image and (right) 2.5D surface with plotted isocontours (lateral perspective of the surface)

As shown in Fig. 4, a 2.5D surface was constructed from each patch based on the average intensity of the RGB channels. Once this surface was obtained, isocontours were plotted on it to highlight regions of equal luminance. Although this representation visually resembles a three-dimensional structure, it actually corresponds to a 2.5D topographic surface in which the height axis (*z*) reflects luminance intensity rather than any physical depth.

In this representation, the areas highlighted in yellow correspond to higher-intensity regions, typically associated with bright lesions such as exudates. In contrast, darker areas are associated with blood vessels and low-contrast lesions such as MAs. Once the topographic surface and its isocontours were generated, an unsupervised KNN-based adaptive filtering step was applied to suppress noise and emphasize clinically relevant features, leveraging local intensity similarities.

Once the patches were processed via adaptive filtering, they were used to perform a statistical similarity analysis to assess their structural consistency.

#### Validation of the coefficient of variation and PCA–K-means clustering

The distribution of the coefficient of variation (CV) was assessed using the Shapiro–Wilk test, yielding a statistic of 0.4483 with *p* < 0.001; thus, the null hypothesis of normality was rejected. Consequently, the CV values show a distribution that is significantly different from normal.

**Fig. 5:**
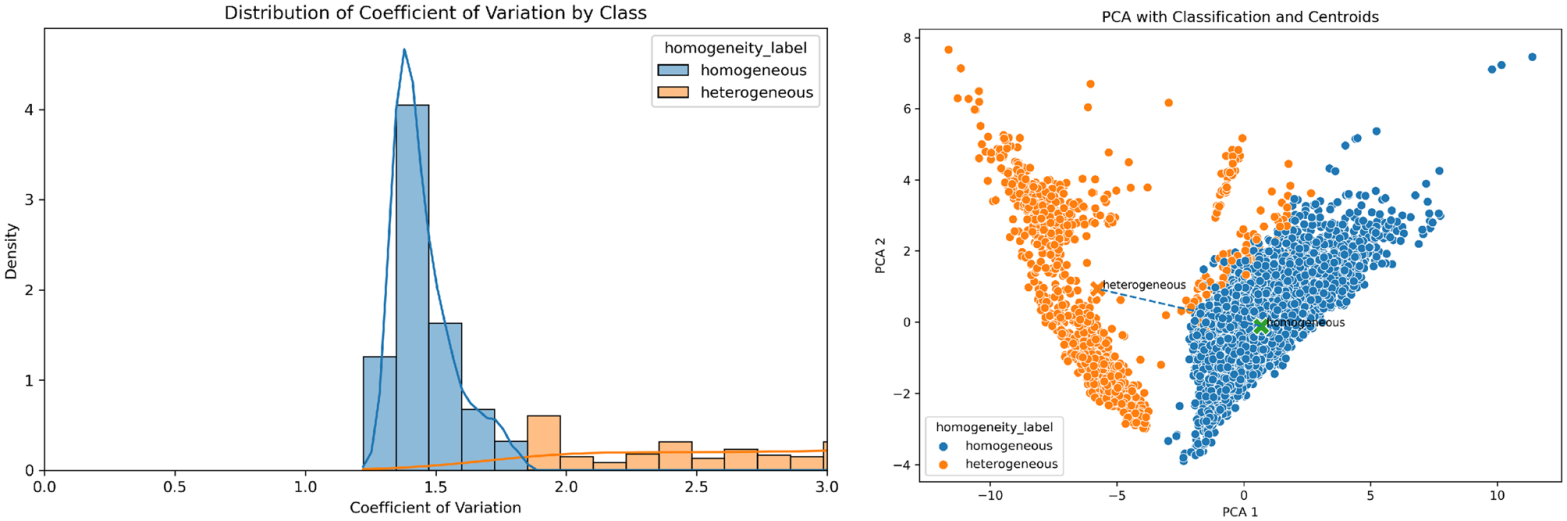
Left: distribution of the CV for image patches by homogeneity label. Right: PCA (PC1–PC2) projection of patches with class assignment (and centroids), stratified by homogeneous vs. heterogeneous label

This behavior can be observed in Fig. 5 (left), which shows the CV distribution for both groups. Homogeneous patches concentrate within a low interval, with a narrow and pronounced peak, indicating low variability. In contrast, heterogeneous patches exhibit a broader distribution shifted toward higher values, with greater dispersion and a right tail, reflecting high internal variability.

To statistically support the classification of patches as homogeneous or heterogeneous, equality of CV variances between groups was evaluated. Levene’s test yielded 17,088.4438 (*p* < 0.001), and Bartlett’s test yielded 21,548.5950 (*p* < 0.001). In both cases, the null hypothesis of equal variances was rejected, indicating a marked difference in CV dispersion.

Accordingly, homogeneous patches concentrate at low and less-dispersed CV values, whereas heterogeneous patches show higher CV values and a wider distribution, supporting the validity of the classification criterion.

Subsequently, to capture patch variability in a multivariate manner, eleven statistical metrics were computed per patch and projected using principal component analysis (PCA). The CV was not used to build the PCA; instead, it was used as a reference to interpret the clusters, such that after applying K-means (*k* = 2) in the PCA space, the cluster with the lowest average CV was assigned as homogeneous. Fig. 5 (right) shows the projection of patches onto the first two principal components and the centroid of each group. As an additional quantitative measure, the Euclidean distance between centroids in the PCA plane (PC1–PC2) was 6.5344, consistent with the clear visual separation between the two groups.

In the PCA space, homogeneous patches (blue points) cluster toward positive PCA1 values, whereas heterogeneous patches (orange points) concentrate toward negative values. The separation between clusters and their centroids supports the ability of the PCA–K-means scheme to distinguish homogeneous from heterogeneous patches.

#### Division of the dataset into homogeneous and heterogeneous patches

As a result of the statistical analysis, a binary classification of patches was established based on homogeneity. Of the 10,213 analyzed patches, 9,125 (89.35%) were labeled as homogeneous and 1,088 (10.65%) as heterogeneous.

From a visual standpoint, homogeneous patches tend to exhibit a consistent appearance: relatively uniform intensities, smooth texture, and gradual changes, with an absence (or minimal presence) of strong edges, artifacts, or abrupt transitions within the crop. In contrast, heterogeneous patches present evident internal variations: more dispersed intensities, areas with abrupt contrast, edges/artifacts (e.g., due to cropping or background), and sudden content changes that break patch uniformity; these characteristics are consistent with the higher CV values observed for this group.

### Hyperparameters and training configuration

Training was conducted for 70 epochs with a batch size of 24 images. The dataset was split into 80% training (7,298 patches) and 20% validation (1,825 patches) using random_split with a fixed seed (SEED = 123). The final reported model corresponds to the checkpoint (.pth) with the highest validation Dice score obtained over the 70 training epochs.

The model was optimized using AdamW  [37] with an initial learning rate of $1 \times 10^{-4}$ and a weight decay of $1 \times 10^{-4}$. AdamW was selected over standard Adam due to its correct implementation of decoupled weight decay, which provides better regularization and generalization compared to the original Adam formulation. This is especially relevant in medical segmentation models with a high number of parameters, such as the one proposed. To adaptively control the learning rate, ReduceLROnPlateau was employed, reducing the rate by a factor of 0.1 when the validation Dice score failed to improve for 4 consecutive epochs. This reduction strategy based on validation performance is a widely adopted standard practice in medical segmentation tasks to prevent stagnation in local minima without requiring a predefined decay schedule.

MA segmentation presents a severe class imbalance, given that lesion pixels represent less than 0.5 to 1% of the total pixels in a retinal image  [38, 39]. In the present dataset (9,123 patches, 1,345,241,088 total pixels), MA pixels accounted for only 0.71% of the total (9,599,916 pixels), confirming an imbalance ratio of 139:1 (background:lesion). To address this, a combined loss function was employed: Focal Loss and Dice Loss.

The loss function used was a combination of Focal Loss and Dice Loss, supported by  [40], who demonstrated that the additive combination of both functions outperforms individual functions in segmentation tasks involving small structures and class imbalance. This choice addresses two inherent challenges in MA segmentation. First, the severe class imbalance between lesion and background pixels, where MA pixels represent only 0.71% of the total pixels in the training dataset, compared to 99.29% corresponding to the retinal background.

Focal Loss (*γ* = 2.0) penalizes incorrectly classified examples with high confidence, forcing the model to focus on MAs that are difficult to distinguish from the background. The gamma = 2.0 factor was adopted following the original recommendation by  [41], later confirmed by  [40], who obtained the best performance with this value among the evaluated options ($\gamma =1, 2, 5, 8$). Dice Loss incorporates a smoothing factor $\varepsilon = 1 \times 10^{-6}$ following the standard practice established by  [42], designed to ensure numerical stability by avoiding division by zero in regions without lesions, making it more robust to class imbalance than standard cross-entropy. Finally, equal weighting ($0.5 \times \mathrm{Focal} + 0.5 \times \mathrm{Dice}$) assigns the same importance to both functions to equitably balance the penalization of difficult examples and the direct optimization of overlap, following the additive combination scheme proposed by  [40].

### Phase I – Base training with DR images

The U-Net was trained directly using only the homogeneous patches selected through the proposed statistical filtering, with the aim of reducing spurious variability associated with heterogeneous patches and stabilizing the optimization process for MA segmentation.

**Fig. 6:**
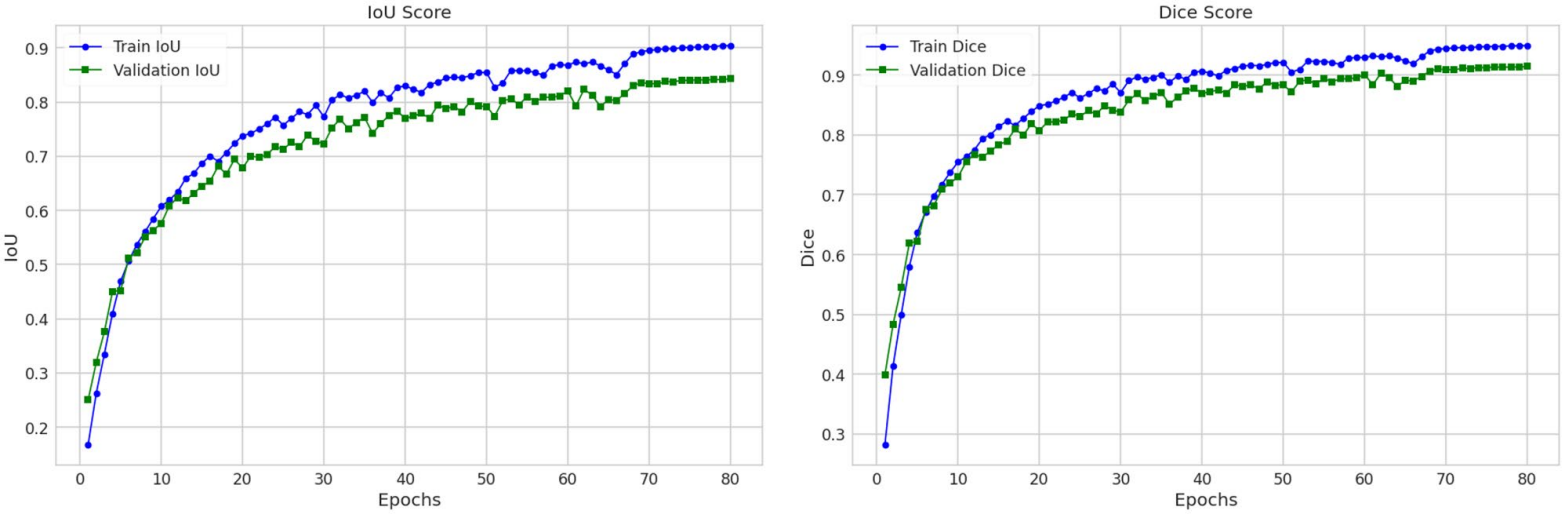
Evolution of IoU and DICE (training and validation) during Phase I for MA segmentation with homogeneous patches

Figure 6 shows the evolution of overlap metrics during training. Both IoU and Dice progressively increase while maintaining a moderate gap between training and validation, suggesting stable learning and adequate generalization. At the best validation point, Dice = 0.9144 and IoU = 0.8429 were obtained.

Overall, these results support the usefulness of the statistical analysis as a preprocessing step to obtain a more stable training set that is less affected by spurious variability, improving MA segmentation performance.

**Fig. 7:**
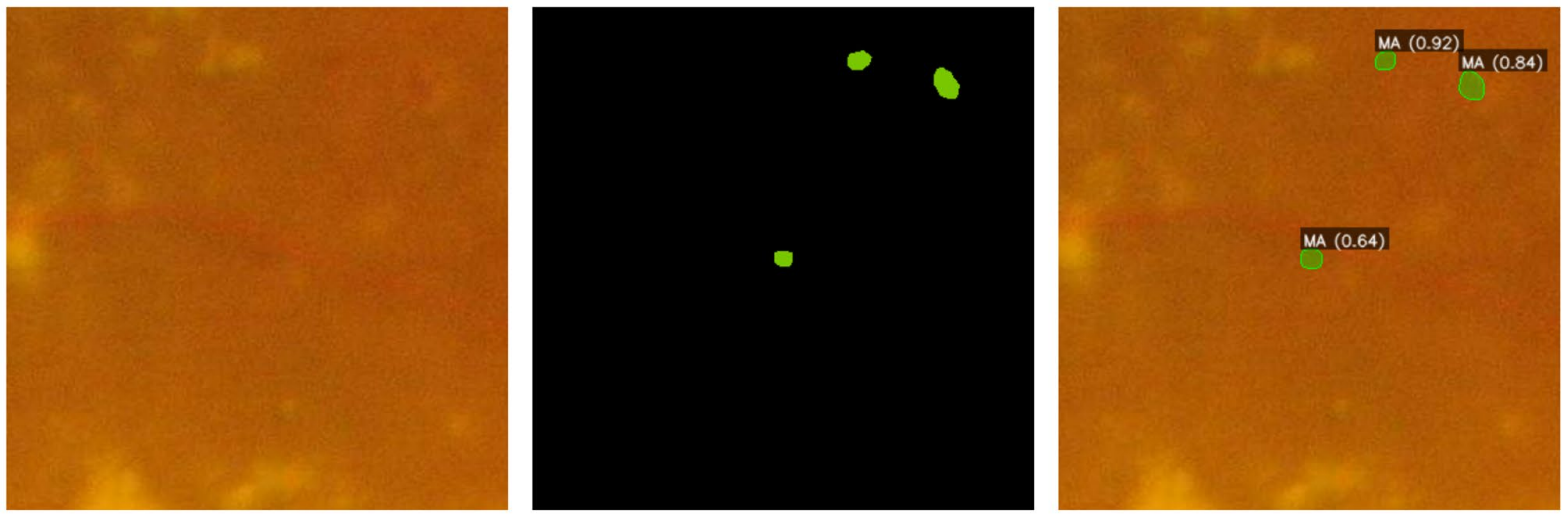
Model evaluation for MA segmentation on DR images: original image (left), reference binary mask (center), and model prediction with IoU values (right)

Figure 7 illustrates an example of the MA semantic segmentation process and its visual evaluation using the U-Net model on DR images.

The third image on the right corresponds to the model prediction. The values overlaid on the predicted image indicate the IoU obtained for each segmented region, evidencing high agreement between the automatic prediction and the reference annotation.

An ablation experiment was conducted comparing two configurations under identical training conditions, Table 3.

**Table 3:** Patch extraction strategies and segmentation performance

Strategy	Method	Patches	Dice	IoU
E1	Lesion-oriented + isocontour	10,213	0.8971	0.8144
E2	E1 + homogeneity filtering	9,123	0.9144	0.8429

E1: lesion-oriented patch extraction with isocontour preprocessing. E2: E1 extended with background homogeneity filtering to remove uninformative patches. Dice and IoU are reported on the validation set

The first configuration (E0—without filtering) utilized the full dataset of 10,213 patches generated through a lesion-oriented strategy with an isocontour channel, achieving a Dice score of 0.8971 and an IoU of 0.8144.

The second configuration (E2—with filtering) applied statistical homogeneity analysis to E1, retaining the 9,123 patches classified as homogeneous via coefficient of variation, Levene–Bartlett tests, and PCA+K-Means clustering, achieving a Dice score of 0.9144 and an IoU of 0.8429 (ΔDice $= +0.0173$, ΔIoU $= +0.0276$).

The results demonstrate that statistical filtering by homogeneity contributes 1.73% points to the final Dice score, validating its inclusion as a preprocessing stage in the proposed pipeline.

To evaluate the contribution of the isocontour channel Table 4, two patch generation strategies were assessed.

**Table 4:** Contribution of the isocontour channel to MA segmentation performance

Strategy	Description	Patches	Dice	IoU
E0	Full coverage (stride 288 px)	10,215	0.5445	0.3842
E1	Lesion-oriented + isocontour	10,213	0.8971	0.8144
E2	E1 + homogeneity filtering	9,123	0.9144	0.8420
Δ (E2 vs E0)			+0.3699	+0.4578

E0: uniform stride-based patch extraction with no lesion guidance. E1: lesion-centered patch extraction with jitter and isocontour preprocessing, no filtering applied. E2: E1 extended with background homogeneity filtering based on coefficient of variation, Levene–Bartlett tests, and PCA+K-Means clustering. Δ denotes the absolute improvement of E2 over E0.

The first strategy (full coverage) generated patches through a uniform stride of 288 px across the entire retinal image, regardless of the presence of lesions, resulting in 10,215 patches and achieving a Dice score of 0.5445 and IoU of 0.3842.

The second strategy (lesion-oriented) generated patches centered on the regions annotated with MAs, applying jitter to increase positional variability, producing 10,213 patches without filtering. After applying isocontour and homogeneity analysis based on the coefficient of variation, Levene–Bartlett tests, and PCA+K-Means clustering, 9,123 patches were selected, achieving a Dice score of 0.9144 and IoU of 0.8420 (ΔDice $= +0.3699$, ΔIoU $= +0.4578$).

The results demonstrate that the lesion-oriented strategy with isocontour-based statistical filtering contributes 36.99% points to the final Dice score compared to full coverage without filtering, validating its inclusion as a preprocessing stage in the proposed pipeline.

### Phase II – (OODG): inference on RL

**Fig. 8:**
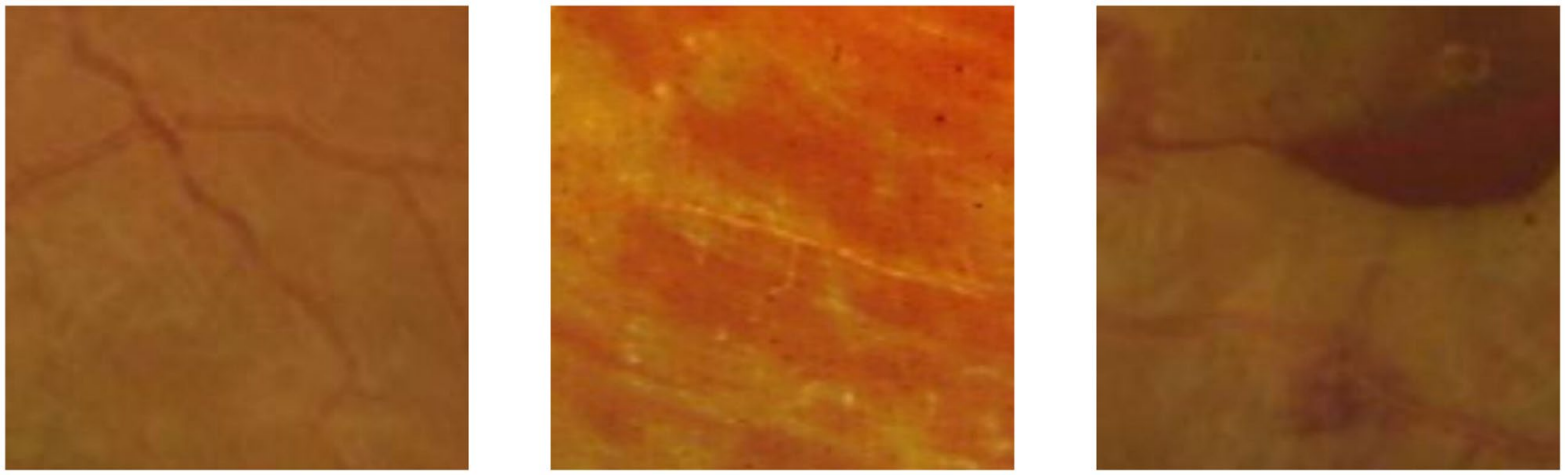
LR patches from a representative fundus image. LR image courtesy of the author, Sashwanthi Mohan MBBS, DNB, FICO  [43]

To evaluate the generalization capability of the U-Net model, inference tests were performed using a set of clinical images corresponding to LR cases that were not used during training. Figure 8 shows an example of a fundus image  [43] split into patches.

**Fig. 9:**
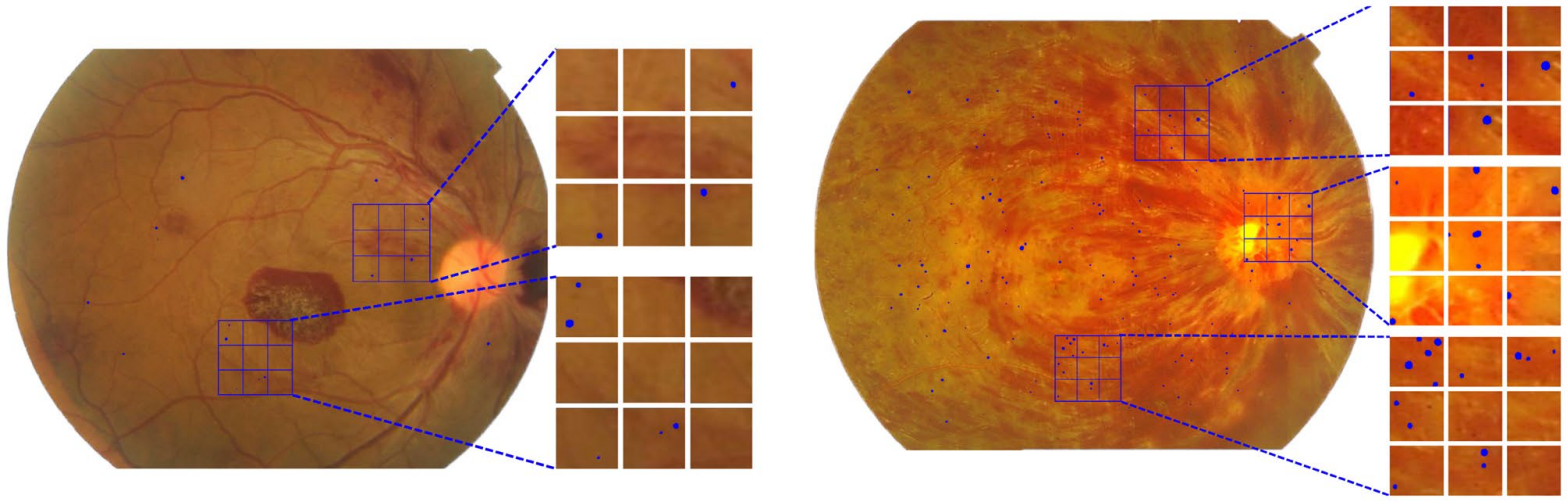
LR patches from a representative fundus image. LR image courtesy of the author, Sashwanthi Mohan MBBS, DNB, FICO.  [43]

Once splitting was completed, the inference process was executed to segment the MAs present in the patches. Figure 9 presents the results of this stage, showing the masks automatically generated by the model as well as the images reconstructed from the processed patches, in which MAs have already been segmented.

In these reconstructions, various pathological manifestations characteristic of LR can be observed, including MAs, which become easier to identify after processing by the proposed model.

### Clinical expert evaluation of segmentation results

Clinical validation was conducted using a structured form. Each specialist was provided with a set of two fundus images containing MA associated with retinopathy. Although several qualitative criteria were considered during the evaluation, they were consolidated into two variables reported in Table 5: Clinical support and MA localization.

**Table 5:** Clinical expert evaluation of MA segmentation

Expert	Clinical support	MA localization
Expert 1	60%	100%
Expert 2	60%	67%
Expert 3	40%	100%
Expert 4	47%	67%

Clinical support was rated on a 1–5 scale and linearly rescaled to 0–100% for quantitative reporting (1→0%, 5→100%). MA localization indicates whether the segmentation aided lesion localization; values are reported as the percentage of “Yes” responses across the evaluated cases

In particular, Clinical support was rated on a 1 to 5 scale and captured the specialist’s perception of the overall clinical value of the segmentation as a visual aid, including how much it helps confirm that the segmented structure corresponds to a MA and the additional benefit it provides for diagnosis or follow-up compared with conventional fundus examination. In turn, MA localization recorded whether the segmentation facilitated MA localization and streamlined inspection during lesion search in each image (Yes/No).

Overall, these two variables, shown in Table 5, provide a quantitative summary of the perceived clinical utility of the proposed segmentations for each expert.

**Fig. 10:**
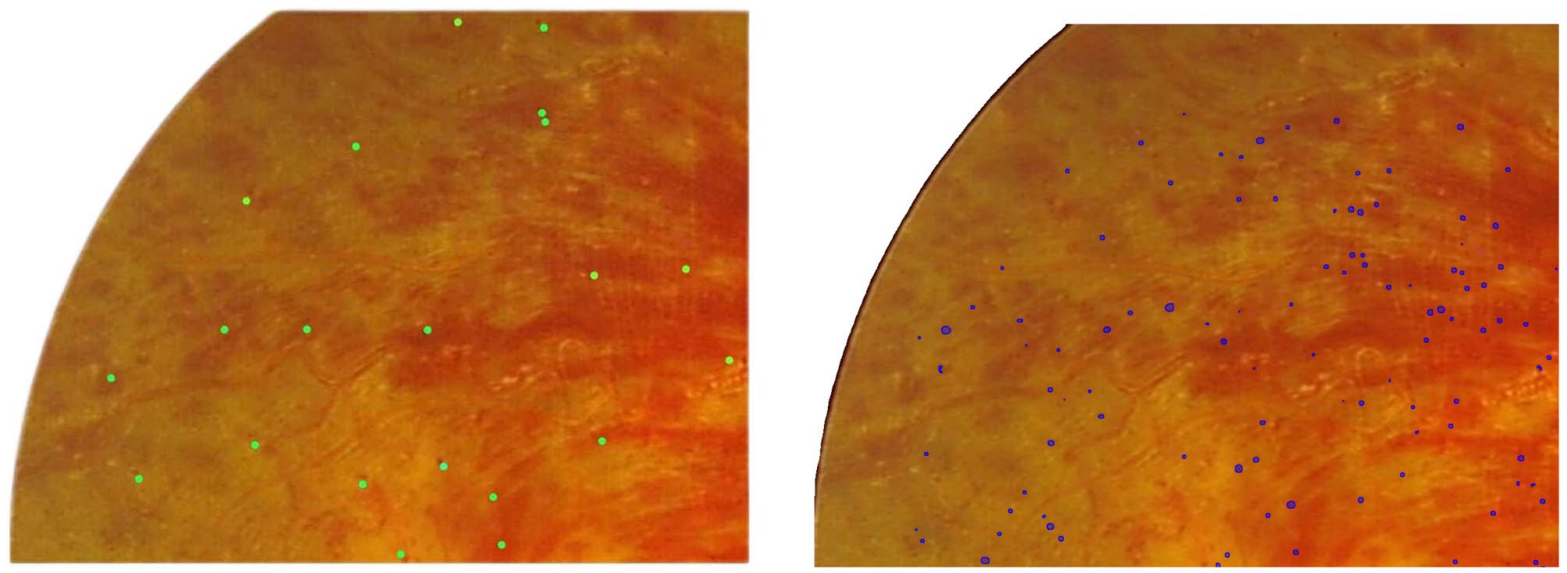
Comparison of annotations in an LR image. (Left) Manual annotations by an ophthalmology specialist (21 identified MAs). (Right) Model detections (102 regions)  [43]

On the other hand, two leukemic retinopathy (LR) images were processed with manual annotation from four ophthalmology specialists per image. To structure the annotation, each image was divided into four quadrants, assigning a specific quadrant to each specialist (Fig. 10). Each specialist annotated independently without access to the others’ annotations, allowing for the quantification of inter-observer variability under controlled conditions.

Image 1: The specialists identified 7, 4, 3, and 4 MAs, respectively (mean 4.5 MAs, SD = 1.5). The model detected 23 regions (5.1x over-detection), with a Precision of 0.1919 (SD = 0.0277), Recall of 0.9286 (SD = 0.1237), and F1-score of 0.3148 (SD = 0.0323).

Image 2: The specialists identified 21, 7, 32, and 11 MAs, respectively (mean 17.8 MAs, SD = 9.7), demonstrating high inter-observer variability attributable to the morphological complexity of lesions in LR. The model detected 102 regions (5.7x over-detection), with a Precision of 0.1694 (SD = 0.1083), Recall of 0.8214 (SD = 0.2342), and F1-score of 0.2716 (SD = 0.1591).

Overall, both images exhibit a consistent pattern: high Recall (0.82 to 0.93) with low Precision (0.17 to 0.19). This indicates that the model preserves its sensitivity for detecting genuine MAs but over-detects other lesions characteristic of LR, such as flame-shaped hemorrhages. It is acknowledged that a more robust evaluation would require a larger set of LR images with pixel-level annotations, which is proposed as future work.

The Fig. 10, illustrates the model’s behavior in the LR domain. Image (left) shows the manual annotations from an ophthalmology specialist (21 identified MAs), while image (right) shows the 102 regions detected by the model. The Recall (0.82) confirms that the model preserves its sensitivity to identify genuine MAs when transferred to the LR domain. The Precision (0.22) reflects an over-detection of 5.7x (102 model vs. 17.8 specialist average), given that lesions such as flame-shaped hemorrhages in the LR domain share morphological and chromatic characteristics with MAs in the DR domain.

This confusion is inherent to the domain shift and does not represent a structural failure of the model, but rather a limitation of operating within a pathology with partially overlapping visual phenotypes.

## Discussion

Generalization across conditions is possible given that: The model’s objective is to segment a morphological phenotype present in both diabetic retinopathy (DR) and leukemic retinopathy (LR), rather than to clinically differentiate between these manifestations.

Microaneurysms (MAs) are a universal anatomical phenotype. This premise is based on the fact that they constitute the initial manifestation and the early biomarker of retinal microvascular damage  [6, 11, 44, 45]. Therefore, regardless of the underlying systemic etiology (as observed in HR  [10], LR  [11], KD  [12], OIS  [13], the lesion exhibits a morphology characterized by: 1) a round-to-oval shape  [28]; 2) a uniform dark red color  [29]; 3) sharp margins  [30]; and 4) localization as protrusions connected to the capillary wall  [31].

The network architecture is specifically designed to localize this well-known morphological pattern (MA).

This biological and technical foundation allows the model to be trained with 7,198 patches and validated with 1,825 patches, corresponding to 81 DR images from the IDRiD dataset. Once the network has internalized the geometric features of MAs, it is capable of identifying these same lesions during the inference phase using 442 patches (221 patches extracted from each of the two leukemic retinopathy images presented). Consequently, by evaluating performance at the patch level rather than the global image level, this sample size (*n* = 442) provides a sufficient quantitative basis to demonstrate the model’s morphological generalization between both conditions.

Table 6 presents a performance comparison across previous works that used U-Net or its variants for microaneurysm segmentation on the IDRiD dataset.

**Table 6:** Comparison of model performance with previous works

Author	Model	IoU	Dice	Accuracy	Loss
**Proposed**	**U-Net**	**0.842**	**0.9144**	0.998	0.120
[20] (2025)	Lightweight Attention U-Net	NR	0.880	0.980	NR
[21] (2025)	CBAM-AG U-Net	0.758	0.865	**0.999**	**0.005**
[24] (2024)	Ens5B-UNet	0.677	0.497	NR	NR
[23] (2024)	R2U-Net	0.449	NR	0.998	NR
[22] (2025)	Modified U-Net	NR	NR	0.998	NR
[46] (2023)	MesU-Net	NR	NR	0.992	0.586

**Bold** indicates the best value per metric (maximum for IoU and Dice). **NR**: Not reported

The proposed U-Net reports an IoU of 0.842 and a Dice score of 0.9144, which are higher than those reported by Tahir et al.  [20] for Dice (0.880) and by Vanaja et al.  [21] for IoU (0.758) and Dice (0.865), for the available metrics. The discrepancies observed in accuracy and loss compared with  [21] may be associated with differences in the training setup (e.g., loss function, class-imbalance handling, data augmentation, as well as preprocessing or postprocessing), in addition to architectural variations and evaluation protocol differences. Overall, the results place the proposed method within the high-performance range reported on IDRiD, particularly in terms of the pixel-wise overlap between the predicted mask and the reference (ground-truth) mask.

On the other hand, the clinical evaluations conducted by four experts (Table 5) indicate that the system offers moderate practical utility. Overall, the model provided an average of 51.8% clinical support and achieved 83.5% MA localization. The specialists described the tool as “very interesting” and considered its purpose “plausible” as a visual aid to guide and accelerate inspection, without replacing medical judgment, emphasizing the relevance of MAs as an early sign of retinopathies, particularly DR.

In this work, the main difference from previous approaches lies in two methodological decisions: (i) training with a subset of patches selected via a statistical homogeneity strategy, discarding highly variable regions that tend to introduce noise, which enabled reducing the amount of training data compared with previous works without degrading overlap metrics; and (ii) evaluating the model under an inter-disease generalization setting by applying it directly, without retraining, to LR, suggesting feasibility in contexts with limited annotation availability. As a complement, the system output was supported through qualitative evaluation by clinical specialists, who scored the segmentations in terms of technical accuracy, confusion severity, clinical support, and MA localization.

OODG in medical imaging presents several challenges, including the following: First is the heterogeneity of the shift: covariate shift (same diagnosis, different device) proves more difficult to manage than semantic shift (different disease) in ophthalmic medical imaging, specifically in fundus images with DR [Ju et al., 2024]. Second is the fragility regarding acquisition changes: a change of scanner within the same modality can cause performance to collapse from a 5.5% error to 46.6%; although partial retraining of the segmentation network allowed recovery up to 3.4%, this highlights that adaptation to new devices is not automatic and requires additional labeled data  [47]. Third is the dependence on semantic context: introducing images of a different disease (DR) as unlabeled data into a glaucoma model degrades performance  [48]. Fourth is the detection of when a model is operating out of distribution, a non-trivial problem in both CNN architectures  [49] and medical vision-language models  [50].

An additional challenge is the scarcity of annotated clinical data for rare retinal pathologies, exacerbated by regulatory and ethical restrictions on the use of ophthalmic images in artificial intelligence models. The use of retrospective patient data for the development of AI algorithms is ethically questioned, as explicit consent is often unfeasible for large historical datasets  [51]. Regulatory frameworks such as the European GDPR classify health data as a special category requiring an explicit legal basis for processing, whether through informed consent or public health interest, imposing requirements for consent and data minimization that restrict the implementation of AI in medical imaging  [52]. This convergence of clinical scarcity and regulatory restriction is particularly relevant for diseases such as leukemic retinopathy, where low prevalence limits the volume of data available for training and validation, making generalization approaches without retraining a viable alternative from a clinical perspective.

## Conclusion

This work presented an inter-disease evaluation scheme, under an out-of-distribution setting, to analyze whether an MA segmenter trained and validated on DR retains its performance when applied directly to LR, without retraining or domain adaptation. By combining a preprocessing strategy based on statistical patch analysis with a U-Net architecture trained on DR, it was possible to detect MAs with acceptable accuracy.

The qualitative validation conducted by specialists suggests that the model has potential as a support tool to highlight relevant patterns and facilitate lesion localization. Although some confusion with visually similar lesions was observed, the experts agreed that this tool can support an initial specialist assessment.

Overall, the findings indicate that knowledge learned from DR can transfer effectively to LR, opening the possibility of applying the approach to identify lesions in diseases with similar lesion structures and patterns. On the one hand, these applications may help reduce diagnostic time and associated complications; on the other hand, they may reduce computational cost, especially in scenarios where annotations and data availability are limited.

## Data Availability

Public dataset: The IDRiD dataset is publicly available via IEEE DataPort: \url{https://ieee-dataport.org/open-access/indian-diabetic-retinopathy-image-dataset-idrid}. Clinical data: LR image courtesy of the author, Sashwanthi Mohan MBBS, DNB, FICO. Images are non-identified with consent for research purposes only.
